# Research prioritization in hernia surgery: a modified Delphi ACHQC and VHOC expert consensus

**DOI:** 10.1007/s10029-024-03139-0

**Published:** 2024-08-27

**Authors:** Daphne Remulla, Mazen R. Al-Mansour, Christopher Schneider, Sharon Phillips, William W. Hope, Joel F. Bradley III, Richard A. Pierce, Luis Arias-Espinosa, Karla Bernardi, Julie L. Holihan, Michelle Loor, Mike K. Liang, Benjamin T. Miller

**Affiliations:** 1 Cleveland Clinic Center for Abdominal Core Health, 9500 Euclid Ave, Cleveland, OH, 44195 USA; 2 Department of Surgery, University of Florida, Gainesville, FL USA; 3 Wright State University School of Medicine, Dayton, OH USA; 4 Department of Biostatistics, Vanderbilt University Medical Center, Nashville, TN USA; 5 Department of Surgery, New Hanover Regional Medical Center, Wilmington, NC USA; 6 Department of Surgery, Vanderbilt University Medical Center, Nashville, TN 37232 USA; 7 Division of General Surgery, NYU Langone, New York, NY USA; 8 Department of Surgery, University of Pittsburgh School of Medicine, Pittsburgh Pennsylvania, USA; 9 Department of Surgery, McGovern Medical School at UTHealth, Houston, TX USA; 10 Michael E. DeBakey Department of Surgery, Baylor College of Medicine, Houston, TX USA; 11 Department of Surgery, HCA Healthcare Kingwood, University of Houston, Kingwood, TX USA

**Keywords:** Research priorities, Hernia surgery, Delphi process

## Abstract

**Purpose:**

Numerous clinical practice guidelines and consensus statements have been published in hernia surgery, however, there is still a need for high-quality evidence to address remaining unanswered questions. The aim of this study was to conduct research priority setting through a modified Delphi process to identify a list of top research priorities in hernia surgery.

**Methods:**

A structured literature review of clinical practice guidelines was performed by the steering committee. Topics considered clinically significant, practical to study and lacking strong evidence were extracted and refined into a comprehensive list, then entered into a two-round Delphi survey for prioritization at the Abdominal Core Health Quality Collaborative (ACHQC) Quality Improvement Summit. In round 1, participants were instructed to select any topic that should be prioritized for future research. Topics were ranked according to the proportion of votes and the 25 highest-ranking topics were included in the second round. In round 2, participants were instructed to select only the top 10 topics for research prioritization.

**Results:**

Eleven clinical practice guidelines were reviewed. Eighty-seven topics were extracted by the steering committee and submitted for prioritization. After the first round, 25 of the highest-ranking topics were determined and included in the second round. A final list of 11 research questions was identified. The hernia types with the most research interest were inguinal and epigastric/umbilical hernias. Other topics of high interest were the management of diastasis recti, primary versus mesh repairs and expectant management versus surgical repair.

**Conclusion:**

Our study provides a research agenda generated through expert consensus that may be used in the prioritization of the design and funding of clinical trials in hernia surgery.

**Supplementary Information:**

The online version contains supplementary material available at 10.1007/s10029-024-03139-0.

## Introduction

Clinical research, particularly randomized controlled trials (RCTs), is the foundation for improving and advancing medical and surgical care. Although the United States Congress increased the National Institute of Health’s base budget by $2 billion to $45 billion in 2022, only $1.3 billion (2.9%) of the total budget was awarded to surgeon scientists for research [1, 2]. With finite resources, research efforts must be carefully prioritized to minimize research waste and produce relevant, high-quality evidence.

Multiple specialty societies, international collaborative groups, and professional organizations have published clinical practice guidelines and consensus statements within hernia surgery [3–5]. However, despite the recommendations put forth in these guidelines and consensus statements, many critical questions remain unanswered. To improve the efficiency and effectiveness of future research, researchers must identify topics to study and questions to answer that will yield the most significant advancements in patient care.

Research priority setting is a process by which a consensus is achieved on research areas or questions of importance to stakeholders [6–8]. The Delphi process has been used extensively in research priority setting across several specialties and involves developing consensus among members through an iterative process [9–11]. The Abdominal Core Health Quality Collaborative (ACHQC) is a collaboration of surgeons whose mission is to maximize the quality and value of health care for patients who suffer from hernia disease [12]. The Ventral Hernia Outcomes Collaborative (VHOC) is a collaboration among hernia surgeons across the United States who share data and develop collective opinions and consensus to help improve the evidence and quality of care for patients with hernias [13–16]. Using a modified Delphi process [17], the ACHQC and VHOC aimed to identify priority research questions in hernia surgery using a consensus-based approach to enable researchers to allocate resources toward these priorities, optimize multi-center collaboration, enhance research impact, and reduce research waste.

## Methods

**Fig. 1:**
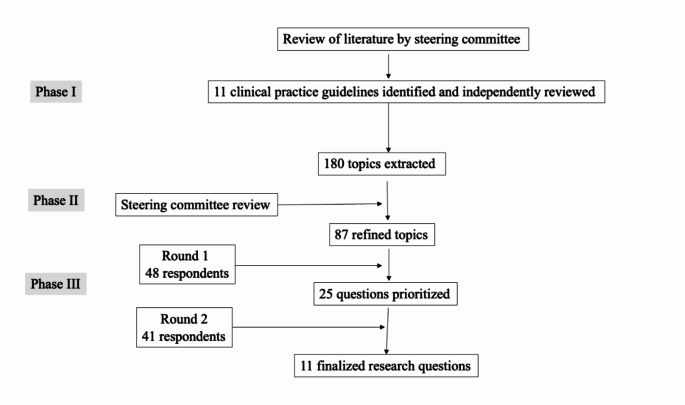
Summary of modified Delphi process

This study implemented a three-phase modified Delphi methodology (Fig. 1). All stages were conducted by expert stakeholders with membership in the ACHQC. Phases II and III were conducted at the 2024 ACHQC Quality Improvement Summit.

### Steering committee

The ACHQC Research Committee served as the steering committee. The ACHQC Research Committee’s purpose is to promote research and evidence-based practice in hernia surgery and to oversee national multi-center prospective studies within the Quality Collaborative [12]. It comprises expert hernia surgeons and biostatisticians from varying clinical practices across the U.S. with membership in the ACHQC.

### Phase I

The steering committee conducted a structured literature review to identify clinical practice guidelines related to hernia surgery. These guidelines were collated and reviewed independently by the ACHQC Research Committee members to identify topics with the following criteria: (1) Lacking strong evidence, (2) Clinically important and (3) Practical to study. Members were asked to record and submit these extracted topics to the chair of the committee (M.A.).

### Phase II

The steering committee convened to review the compiled list of extracted topics. These topics were refined to eliminate duplicates and consolidate similar topics to generate a concise list. These topics were subsequently transcribed into a survey in the Research Electronic Data Capture (REDCap®) web application.

### Phase III

An in-person, two-round modified Delphi consensus method was performed to identify the top research priorities at the 2024 ACHQC Quality Improvement Summit. The Delphi technique is a structured communication method used to obtain consensus among a panel of experts through iterative rounds of questionnaires [17]. Our consensus determination was based on the topics that received the highest proportion of votes in each round. This voting-based approach allowed us to objectively identify the research topics that garnered the most support among our expert panel. The surveys were distributed via a secure REDCap® link. Results were managed electronically using Excel. Each round was conducted over a 24-hour period. Participation was anonymous, and respondents were able to discuss topics between rounds. In round 1, participants were instructed to select any number of topics they felt were important topics for future research in hernia surgery. Topics were ranked according to the proportion of votes. The 25 highest-ranking topics were considered to have reached consensus and included in the second round. In round 2, participants were instructed to select only the 10 topics of highest importance. We defined consensus as the top 10 ranked topics.

## Results

A summary of the modified Delphi process is shown. Eleven clinical practice guidelines were reviewed, from which 87 topics were extracted and published in a REDCap survey. Forty-eight participants completed Round 1, and 41 participants completed Round 2. There was a tie for #10, therefore 11 total research questions are included (Table 1). The hernia types displaying the greatest amount of research interest were inguinal hernias (three questions) and epigastric/umbilical hernias (three questions). Diastasis recti was a prevalent theme highlighted in three questions, two of which involved concomitant hernia and rectus diastasis repair. Questions related to management strategies were also emphasized in the finalized list, particularly with respect to primary versus mesh repair (two questions) and expectant management versus surgical repair (two questions).

**Table 1:** Top 11 research questions

1. Activity restriction versus no restriction following incisional hernia repair
2. Does concomitant diastasis repair reduce recurrence of umbilical/epigastric hernias?
3. Effect of repair of contralateral occult inguinal hernia found at the time of minimally invasive groin repair on quality of life
4. Rate of long-term bowel related outcomes of intraperitoneal mesh
5. Outcomes of neurectomy and/or mesh removal for chronic post-operative inguinal pain using a control group
6. Safety of synthetic mesh during emergent inguinal hernia repair with bowel resection for strangulation
7. Outcomes of suture versus mesh repair for umbilical/epigastric hernias with concomitant diastasis
8. Expectant management versus surgical repair for parastomal hernias
9. Plication/suture repair versus mesh repair for diastasis recti
10. The role of robotic surgery for umbilical and epigastric hernia repair
11. Expectant management for contralateral occult inguinal hernia found at the time of minimally invasive groin repair

## Discussion

This study identified the top 11 contemporary, clinically relevant research topics in hernia surgery through a modified Delphi consensus methodology. Our results provide a research agenda derived from an expert consensus between the ACHQC and VHOC, two national organizations dedicated to the delivery of high-quality patient care through research and evidence-based practices, that may be used in the prioritization of the design and funding of clinical trials in hernia surgery.

The primary aim of research priority setting is to identify the most relevant and feasible areas of study in order to optimize the use of resources and generate prospective clinical studies that will most meaningfully impact patient management and care. Critical components of research prioritization include clearly defined priority criteria, a systematic and transparent methodology, and engagement of key stakeholders [6]. While there is no gold standard approach, we used a modified Delphi process which has been successfully utilized in prior research prioritization work identifying the top 10 research priorities in varying specialties [9–11].

Scrimgeor et al. published research prioritization in hernia surgery in 2022. In this study, the Scottish Surgical Research Group and the British Hernia Society conducted a modified Delphi process across 16 countries and six continents with a broad group of stakeholders including patients, healthcare professionals, professors, and surgical trainees. Their results highlight 14 research priorities in hernia surgery [18]. Interestingly, a predominant theme in their results was an emphasis on defining outcome measures. For example, three priority questions were: “What are the most important patient outcomes following hernia surgery?”, “What are the most important outcome measures for incisional hernia repairs?” and “What are the optimal outcome measures following hernia surgery?”. Another theme illustrated by their findings was the need to clarify the incidence, etiology, and prevention strategies of chronic pain after hernia surgery [18]. Significant differences exist between the results of the current study and that of Scrimgeor et al. We found that our research priorities focused primarily on exploring, rather than defining, outcomes between groups with different management strategies. For example, two questions involved comparing suture/plication versus mesh repair for patients with diastasis recti (#7, #9), while another involved comparing concomitant diastasis repair to hernia repair alone (#2). Another theme was the comparison of outcomes in patients managed with an expectant management strategy versus surgical repair for inguinal (#3) and parastomal hernias (#8). These differences in our findings may be largely attributed to the variations in our methodologies. Firstly, our research questions were generated using a structured review of published clinical guidelines. In contrast, Scrimgeor et al. generated research questions using individual viewpoints submitted by stakeholders. Furthermore, our panel was restricted to hernia surgeons practicing in the U.S., while Scrimgeor et al. recruited stakeholders of different professions and experience levels, as well as members of the public using a dedicated Twitter^®^ account. It is therefore difficult to ascertain whether this disparity is a result of differing research priorities globally or due to the composition of panel members and their respective familiarity with current evidence. While the ACHQC is comprised of diverse members with regard to practice setting, years in practice, and gender, all members, and therefore the entirety of our panel, are currently practicing hernia surgeons. As such, it follows that research questions from this panel would be more specific to the management of commonly encountered clinical scenarios compared to a broader panel.

Ultimately, by comparing the results of research prioritization across diverse contexts, we are able to underscore topics of particular importance. Both our work and that of Scrimgeor et al. identified the impact of different types of mesh, long-term outcomes of intraperitoneal mesh repairs, and strategies for the management of chronic groin pain as topics with a current need for future research. Similarly, by juxtaposing previous research prioritization topics with contemporary ones, questions that have remained unanswered are brought forth. In 2014, Stefanidis et al. published 40 research topics in gastrointestinal and endoscopic surgery, of which six questions were related to hernias [19]. Over the past decade and into the present, there remains a demand for evidence on the outcomes of primary versus mesh repairs, the safety of permanent mesh in contaminated procedures, and the quality-of-life benefit following hernia repair. The recognition of these shared research priorities enables researchers to maximize research impact via the funding and design of multi-center clinical trials that address both the most current pressing research questions and those that have remained unresolved.

Before studies can be designed to undertake these research initiatives, it is important to highlight clinical research that is currently underway to minimize research waste and maximize efficiency. Two studies are presently ongoing that may offer valuable insight on occult inguinal hernias and the questions posed by priorities #3 and #11. An RCT comparing upfront surgical repair to the expectant management of occult inguinal hernias is currently recruiting patients with the aim of developing a decision tool and comparing outcomes (NCT04815707). Additionally, we await the results of a 15-year observational cohort study of 201 patients with occult ventral and inguinal hernias that may provide a greater understanding of the natural course of expectant management in these patients (NCT04683367). Lastly, research priority #10 may be addressed by a recently initiated RCT by Nielsen et al. for patients with primary ventral hernias who will be randomized to undergo an open or robotic repair, which may better our understanding of the role of robotic surgery in these cases (NCT05906017).

Several limitations in this study exist. Firstly, there is an absence of non-physician stakeholders. We intentionally chose to recruit hernia surgeon stakeholders to optimize collaboration and coordination among surgeon researchers who are familiar with the interpretation, design, and execution of multi-center research studies. However, in doing so, we excluded the patient perspective. Future efforts should aim to engage a more inclusive group of stakeholders to ensure research priorities reflect the needs and perspectives of the broader community and enhance research impact. It is critical for future research prioritization studies to consider patient values and concerns throughout the surgical process as patient-informed research agendas may steer research towards areas with the highest potential for improving patient experiences and outcomes.

Furthermore, we exclusively recruited participants from the ACHQC who attended the 2024 Quality Improvement Summit. Many general surgeons in the U.S. perform hernia surgery and are not affiliated with the ACHQC, and their interests may not be well-represented in this study. We acknowledge that our findings primarily provide insights into the U.S. perspective on hernia surgery research priorities, which may differ from other countries due to varying technical practices and healthcare systems.

Despite these limitations, we achieved a diverse panel of hernia experts representing different clinical practice settings and national geographical distribution with high retention (85.4%) between rounds. Furthermore, our methodology was transparent, simple to execute and highly reproducible. While specific to the U.S. context, our findings offer valuable insights into research priorities in hernia surgery and may serve as a benchmark for comparative studies in other countries.

## Conclusion

In conclusion, research priority setting in hernia surgery plays a critical role in shaping the direction of future research. Our study highlights 11 high-priority research topics that can be used to address the needs of stakeholders through the promotion of multi-center collaboration and the production of meaningful, high-quality evidence.

## Electronic supplementary material

Below is the link to the electronic supplementary material.

Supplementary Material 1

## Data Availability

We do not plan to make data publicly available.

## Abbreviations

ACHQC: Abdominal Core Health Quality Collaborative
VHOC: Ventral Hernia Outcomes Collaborative
REDCap: Research Electronic Data Capture
