# Supplementary material for: Research prioritization in hernia surgery: a modified Delphi ACHQC and VHOC expert consensus
Source: Hernia. 2024 Aug 27;28(6):2217–22. doi: 10.1007/s10029-024-03139-0 (PMC11530562; doi:10.1007/s10029-024-03139-0)
Supplement: Supplementary file 1 — Supplementary Material 1 [file 10029_2024_3139_MOESM1_ESM.docx]

**Statements and Declarations**:

Funding: This study received no funding.

Competing Interests:

Daphne Remulla, Luis Arias-Espinosa, Sharon Phillips, Christopher Schneider, Julie L. Holihan, Joel F. Bradley III, Michelle Loor, and Karla Bernardi have no disclosures.

Mazen R. Al-Mansour receives speaker fees from Medtronic, consultation fees from Abbvie Inc., educational payments from Intuitive Surgical Inc., and food and beverage from W.L. Gore & Associates.

William W. Hope receives honorarium for consulting/research support/Speaking from BD and W.L. Gore & Associates, honorarium for consulting/research support from Allergan, Intuitive Surgical and Medtronic.

Richard A Pierce receives research support from Intuitive Surgical Solutions, Inc., Cook Biotech and Bard/Davol. His spouse is an employee at BD within the CareFusion Division.

Mike K. Liang serves as an expert consultant for mass tort legal cases.

Benjamin T. Miller Benjamin Miller MD received a research grant from the American Hernia Society and Integra LifeSciences.

Data Access Statement:

We do not plan to make data publicly available.

Ethical Statements:

This study is a consensus statement between two professional societies. This study did not involve patients and therefore did not require informed consent.
